# Cost-Analysis of Seven Nosocomial Outbreaks in an Academic Hospital

**DOI:** 10.1371/journal.pone.0149226

**Published:** 2016-02-10

**Authors:** Jan-Willem H. Dik, Ariane G. Dinkelacker, Pepijn Vemer, Jerome R. Lo-Ten-Foe, Mariëtte Lokate, Bhanu Sinha, Alex W. Friedrich, Maarten J. Postma

**Affiliations:** 1 Department of Medical Microbiology, University of Groningen, University Medical Center Groningen, Groningen, the Netherlands; 2 Department of Medical Microbiology, University Hospital Tübingen, Tübingen, Germany; 3 Department of Pharmacy, Unit of PharmacoEpidemiology & PharmacoEconomics, University of Groningen, Groningen, the Netherlands; 4 Institute of Science in Healthy Aging & healthcaRE (SHARE), University Medical Center Groningen, Groningen, the Netherlands; 5 Department of Epidemiology, University Medical Center Groningen, Groningen, the Netherlands; Hôpital Robert Debré, FRANCE

## Abstract

**Objectives:**

Nosocomial outbreaks, especially with (multi-)resistant microorganisms, are a major problem for health care institutions. They can cause morbidity and mortality for patients and controlling these costs substantial amounts of funds and resources. However, how much is unclear. This study sets out to provide a comparable overview of the costs of multiple outbreaks in a single academic hospital in the Netherlands.

**Methods:**

Based on interviews with the involved staff, multiple databases and stored records from the Infection Prevention Division all actions undertaken, extra staff employment, use of resources, bed-occupancy rates, and other miscellaneous cost drivers during different outbreaks were scored and quantified into Euros. This led to total costs per outbreak and an estimated average cost per positive patient per outbreak day.

**Results:**

Seven outbreaks that occurred between 2012 and 2014 in the hospital were evaluated. Total costs for the hospital ranged between €10,778 and €356,754. Costs per positive patient per outbreak day, ranged between €10 and €1,369 (95% CI: €49-€1,042), with a mean of €546 and a median of €519. Majority of the costs (50%) were made because of closed beds.

**Conclusions:**

This analysis is the first to give a comparable overview of various outbreaks, caused by different microorganisms, in the same hospital and all analyzed with the same method. It shows a large variation within the average costs due to different factors (e.g. closure of wards, type of ward). All outbreaks however cost considerable amounts of efforts and money (up to €356,754), including missed revenue and control measures.

## Introduction

Nosocomial outbreaks are a major problem for health care institutions due to increased morbidity and mortality for the affected patients. The containment and control of these outbreaks costs substantial amounts of funds and resources, especially when left unnoticed or untreated [1]. Rising antimicrobial resistance levels further increase the difficulty to treat nosocomial infections, incurring increasing costs [2–4]. Although it is known for some organisms what the burden of disease is when a nosocomial infection occurs [3–5], estimates of the exact costs for health care institutions during outbreaks are scarce. Knowing the average cost of an outbreak per patient per day can help decision makers to justify the necessary investments in infection prevention and control measures, thus improving the decision making process[6]. This study sets out to evaluate several nosocomial outbreaks within a single Dutch academic hospital with an active Infection Prevention Unit, over a time period of three years.

Within the Netherlands there is proactive national infection prevention and control collaboration through the Working group Infection Prevention (www.wip.nl). They provide over 130 different guidelines on infection prevention, stating all the actions health care institutions should perform and facilitate. The Search-and-Destroy policy for MRSA is one of the success stories of the Dutch infection prevention approach [7]. In this study we provide a transparent cost-analysis, describing in detail the costs that occur during the control of an outbreak in a large Dutch academic hospital and related costs of missed revenues due to closed beds. These data will give a comparable overview of outbreaks caused by multiple microorganisms in one health care center, thus providing novel insights into nosocomial outbreak costs.

## Material and Methods

All evaluated outbreaks occurred between 2012 and 2014 in a university medical center in the north of the Netherlands with 1339 registered beds, including a separate rehabilitation center.

Outbreaks for which all data was available to perform an analysis were evaluated. Costing was done from a hospital perspective. All identifiable extra costs that were made due to an outbreak were taken into account (from the start of the outbreak until one year after the end of the outbreak). An outbreak was defined as at least two patients who were tested positive as indicator for colonization or infection for the same microorganism (bacterial or viral), with some epidemiological link (e.g. same time-period, same ward). The duration of an outbreak was counted in days and began on the day that the Infection Prevention Unit started measures to control the outbreak until the day that they decided the risk of transmission was over and additional control measures were not deemed necessary anymore. When an outbreak was suspected, the Infection Prevention Unit provided assistance to the affected ward and advised on extra surveillance cultures, extra cleaning, isolation of patients, and possible closure of the ward if necessary. Actions are based on the local and national infection prevention guidelines.

Counted costs were divided into: microbiological diagnostics/surveillance costs; missed revenue due to closed beds (based upon the difference in bed occupancy rates compared to the two months before); additional cleaning costs; additional personnel (infection prevention, nursing staff and clinicians); costs made for contact or strict isolation of patients and other costs (e.g. purchase of extra materials, possible prolonged length of stay, extra medication). In order to take into account all possible costs that occurred, a wide range of different sources were used to ensure that no expenses were overlooked. Admission data came from the general hospital database. Numbers of cultures came from the Medical Microbiology Database and the prices for the diagnostics were internal cost prices (depending on type of diagnostic between €25 and €200 per sample). Extra personnel and possible other costs were calculated based on interviews with the, at that time, involved staff (i.e. head nurses and medical specialists), together with detailed case reports from each outbreak made by the Infection Prevention Unit. Bed day cost prices (ranging between €464.39 and €1829.78, depending on the type of ward and excluding variable costs) and personnel costs (ranging between €19.23 and €75.54 per hour) were based upon Dutch reference prices [8]. It was assumed that the hospital will not lay off any (temporary) personnel during closure of wards, meaning personnel costs were considered fixed for this study. When calculating extra workload for the personnel due to infection control measures, these costs were included as opportunity costs. Isolation costs were calculated after internal evaluation (€25.14 for contact and €33.51 for strict isolation per day). Additional cleaning costs and the prices for those were calculated based on interviews and stored records as provided by the department of technical—and facility services (€25.38 per hour). When calculating the missed revenue, only the (fixed) bed day cost price was taken into account. Possible opportunity costs for the (fixed) personnel costs were left out in order to be more conservative in the calculation. All prices were converted to 2015 Euro level, using Dutch consumer index figures (www.cbs.nl).

This analysis followed the CHEERS guideline and included all applicable items as recommended when reporting economic evaluations [9]. The study was purely observational and retrospective of nature and performed on outbreak level. The anonymized data used for the analyses were collected by those authors functioning as treating physicians, from the department’s own database. The collected data did not include any (in)directly identifiable personal details and the analyzing authors had no access to those, complying with the local data protection committee regarding clinical data processing. Following Dutch legislation and guidelines of the local ethics commission approval was therefore not required (http://www.ccmo.nl). Calculations were done with Microsoft Excel (Microsoft, Redmond, WA, USA) and SPSS (IBM, Amonk, NY, USA). When statistics were performed, a significance level of p < 0.05 was applied.

## Results

### Outbreak and patient characteristics

In total, seven different outbreaks could be financially evaluated. One outbreak caused by a virus and seven bacterial outbreaks (see Table 1 for the responsible microorganisms). For these outbreaks there were between 3 and 37 positive patients. The duration was between 16 and 86 days. Characteristics of the outbreaks can be found in Table 1. The *Pantoea* transmission was treated as an outbreak because it occurred on a neonatal ICU.

**Table 1:** Outbreak and patient characteristics.

Microorganism	Year	Ward	Positive persons	Average age (years)	Gender(% male)	Duration (days)
*Pantoea* spp.	2012	ICU	9	0.0 (25 days)	54%	36
*S*. *aureus* (MRSA)	2012	Nursing	3	56.9	75%	16
*K*. *pneumonia* (ESBL)	2012	Nursing	5	73.2	50%	24
*K*. *pneumonia* (ESBL)	2012	Rehabilitation	9	44.5	100%	17
*E*. *faecium* (VRE)	2013	Nursing	19	61.1	95%	50
Norovirus	2013	Rehabilitation	37	59.6	53%	28
*S*. *marcescens*	2014	ICU	8	0.1	50%	86

MRSA: Methicillin-resistant *Staphylococcus aureus*; ESBL: Extended-spectrum beta-lactamase; VRE: Vancomycin-resistant *Enterococcus*.

### Cost-analysis

**Fig 1:**
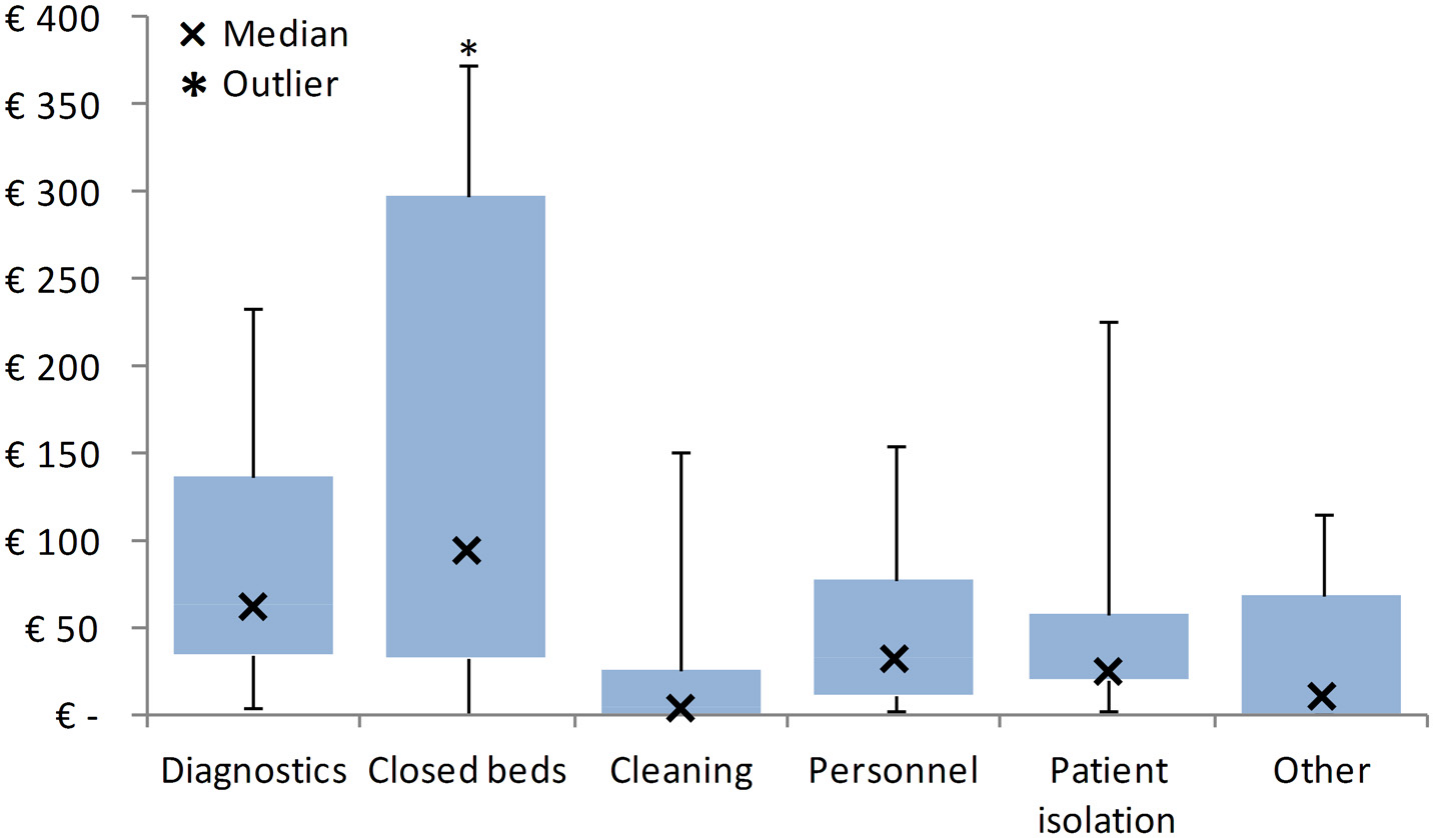
Interquartile ranges of the costs (in Euros) per category. Medians are depicted by the X, within the closed beds there was one outlier of €1144.

Total costs for the hospital ranged between €10,778 for the Norovirus outbreak and €356,754 for the 2014 *S*. *marcescens* outbreak. On average, costs per positive patient per outbreak day, ranged between €10 for the Norovirus outbreak and €1,368 for the ESBL *K*. *pneumonia* on the nursing ward (95% CI: €49-€1042). The mean of the total average costs per positive patient per day comes to €546 and the median to €519.Within the average costs per positive patient per outbreak day, the majority of the costs (50%) were made because there was the closure of (multiple) ward(s) leading to missed revenue; 17% of the costs were for extra microbiological diagnostics; 11% due to contact or strict isolation of patients; 10% for extra personnel; 7% for other costs; and 5% due to extra cleaning on the affected wards (see Table 2). Interquartile ranges of the costs per different categories are displayed in Fig 1. Binary regression analysis showed that a bacterial outbreak is correlated with higher average costs per patient per day compared to the single viral outbreak (p = 0.02).

**Table 2:** Average costs per positive patient per outbreak day.

Microorganism	Total	Diagnostics	Closed bed	Cleaning	Personnel	Patient isolation	Other
*Pantoea* spp.	€88.11	€53.50	€0.00	€ 0.70	€8.80	€ 24.40	€0.70
*S*. *aureus* (MRSA)	€657.08	€205.20	€221.79	€34.67	€ 112.33	€ 72.25	€ 10.83
*K*. *pneumonia* (ESBL)	€1,368.92	€70.59	€ 1,144.50	€17.76	€33.27	€ 46.28	€ 56.52
*K*. *pneumonia* (ESBL)	€980.51	€234.17	€ 98.03	€150.93	€ 154.94	€ 227.55	€ 114.90
*E*. *faecium* (VRE)	€197.26	€ 64.45	€ 69.27	€ 3.74	€42.08	€ 17.72	€0.00
Norovirus	€10.40	€ 5.21	€0.00	€ 0.55	€2.21	€2.33	€ 0.10
*S*. *marcescens*	€518.54	€ 19.34	€375.00	€ 0.68	€15.59	€ 25.67	€ 82.27

MRSA: Methicillin-resistant Staphylococcus aureus; ESBL: Extended-spectrum beta-lactamase; VRE: Vancomycin-resistant Enterococcus.

## Discussion

Seven outbreaks were evaluated and costs varied substantially. On average, the additional costs due to an outbreak were €546 per positive patient per outbreak day. These average costs ranged between €10 and €1,369. The most expensive outbreak per patient per outbreak day was an ESBL producing *K*. *pneumonia*. The highest costs in this outbreak occurred due to a two week closure of the ward, which not only led to a drop in admitted patients, but also to a cancellation of scheduled procedures which could not be replaced with others due to the short term of the cancellation. The lowest average costs were made for the Norovirus outbreak. This was mainly due to the specifics of this infection, less diagnostics were necessary, because only patients with Noro-like symptoms (e.g. watery diarrhea) were tested. Due to the short incubation time, chances of undetected transmission are small. Furthermore, in this case closure of the ward was deemed unnecessary and there was no excess morbidity or mortality for the patients due to their Noro infection. In almost all cases of the evaluated outbreaks, patients were colonized but not infected. During the *Klebsiella* outbreak in the rehabilitation center there was excess morbidity in the form of one sepsis episode and subsequently all costs made during this admission were taken into account and categorized under ‘Other’. For the *S*. *marcescens* outbreak there was excess length of stay observed for two patients by the treating medical specialist. Also in this case, these costs were taken into account and categorized under ‘Other’. Ergo, it seems that outbreak and microorganism specific characteristics cause large variation in the total costs. This variation together with the relatively small number of outbreaks also meant that correlation analyses on the data were impracticable. We therefore chose to give a descriptive overview. Based upon this cost-analysis we hypothesize that on average a viral outbreak is most likely to be less expensive than a bacterial one. This is mainly due to easier and quicker detection which reduces the duration and the microbiological costs. For bacterial outbreaks we observed large variation in the costs. One of the biggest cost drivers is the closure of wards and the subsequent drop in revenue, especially when this closure has consequences for scheduled procedures. Cleaning costs are dependent on the type of ward, with less additional costs on an ICU ward, because cleaning procedures are already strict and higher costs in the rehabilitation center due to extra rooms (e.g. physiotherapy exercise rooms) that had to be cleaned more strict than normal.

Although there are numerous studies on the (financial) burden of disease of resistant organisms and nosocomial infections [3, 10], there are just a few cost-analyses published on nosocomial outbreaks. Notably, financial evaluations of Norovirus outbreaks are published most [11–15]. Besides Noro, there are publications on *P*. *aeruginosa*, MRSA, Acinetobacter and VRE [16–19]. Although difficult to compare, because the methods were not always similar, total costs seem comparable, but average costs per patient per outbreak day seem slightly lower than those found here. The difference is likely to be caused by different definitions for the duration of an outbreak. This study took the time during which extra infection prevention measures on the ward were in place. Others choose to count the days between the first positive culture of the index patient until discharge of the last positive patient, which might be considerably longer, thereby lowering the average cost. The lack of financial evaluations and consistency within those evaluations does however clearly show the need for more studies and especially a more consistent methodological approach.

Strength of this study is the fact that multiple outbreaks caused by different microorganisms in a single hospital were evaluated. This gives a comparable overview of how cost categories differ between different outbreak situations. Limitations are that it is a retrospective analysis and it might be that data are missing because of this. However, by using multiple sources for data, this aspect is minimized. Still, preferable all data are collected prospectively. Depending on national health care systems it will differ who will be bear the costs. This makes comparison between different studies from different countries more difficult. By presenting the costs within different categories we tried to make interpretation of the data more flexible and adaptable to other settings. Finally, with only one viral outbreak, this category is under represented, there were however no more suitable viral outbreaks to include during the evaluated time-period.

Concluding, we present a cost-analysis of multiple outbreaks in an academic center. Although costs differ between different outbreaks, due the microorganism or type of ward, the average costs per patient per day seem substantial. Especially with ever rising antimicrobial resistance levels, such outbreaks as described here are becoming continuously more difficult to treat and costs are expected to rise even further in the future. Average costs of an outbreak per patient per day as presented here can be used to further clarify costs and benefits within hospitals related to infection prevention. Ultimately this should help decision makers to justify the necessary investments in infection prevention and control measures. Our study may thus contribute to more transparency in health care budgets and improve the decision making processes concerning where to invest. Notably, extra argumentation can be found in these findings for investments into infection prevention and control measures to avert outbreaks or to contain them swiftly.
